# Real-Time Dual-Wavelength Time-Resolved Diffuse Optical Tomography System for Functional Brain Imaging Based on Probe-Hosted Silicon Photomultipliers

**DOI:** 10.3390/s20102815

**Published:** 2020-05-15

**Authors:** David Orive-Miguel, Laura Di Sieno, Anurag Behera, Edoardo Ferocino, Davide Contini, Laurent Condat, Lionel Hervé, Jérôme Mars, Alessandro Torricelli, Antonio Pifferi, Alberto Dalla Mora

**Affiliations:** 1 CEA, LETI, MINATEC Campus, F-38054 Grenoble, France; lionel.herve@cea.fr; 2 University Grenoble Alpes, CNRS, Grenoble INP, GIPSA-Lab, 38000 Grenoble, France; jerome.mars@gipsa-lab.grenoble-inp.fr; 3 Politecnico di Milano, Dipartimento di Fisica, 20133 Milano, Italy; anurag.behera@polimi.it (A.B.); edoardo.ferocino@polimi.it (E.F.); davide.contini@polimi.it (D.C.); alessandro.torricelli@polimi.it (A.T.); antonio.pifferi@polimi.it (A.P.); alberto.dallamora@polimi.it (A.D.M.); 4 Visual Computing Center, King Abdullah University of Science and Technology (KAUST), Thuwal 23955-6900, Saudi Arabia; laurent.condat@kaust.edu.sa; 5 Consiglio Nazionale delle Ricerche, Istituto di Fotonica e Nanotecnologie, 20133 Milano, Italy

**Keywords:** diffuse optical tomography, time-correlated single-photon counting, silicon photomultipliers, functional imaging

## Abstract

Near-infrared diffuse optical tomography is a non-invasive photonics-based imaging technology suited to functional brain imaging applications. Recent developments have proved that it is possible to build a compact time-domain diffuse optical tomography system based on silicon photomultipliers (SiPM) detectors. The system presented in this paper was equipped with the same eight SiPM probe-hosted detectors, but was upgraded with six injection fibers to shine the sample at several points. Moreover, an automatic switch was included enabling a complete measurement to be performed in less than one second. Further, the system was provided with a dual-wavelength (670 nm and 820 nm) light source to quantify the oxy- and deoxy-hemoglobin concentration evolution in the tissue. This novel system was challenged against a solid phantom experiment, and two in-vivo tests, namely arm occlusion and motor cortex brain activation. The results show that the tomographic system makes it possible to follow the evolution of brain activation over time with a 1s-resolution.

## 1. Introduction

Near-infrared diffuse optical tomography (DOT) is a non-invasive photonics-based imaging technique for biomedical applications. It has been used in different fields such as functional brain imaging [1,2], breast cancer analysis [3,4], small-animal imaging [5] and flaps monitoring [6,7,8] just to mention a few. The spatial resolution obtained with DOT is low as compared to other medical imaging modalities, such as functional magnetic resonance imaging (fMRI) or positron emission tomography (PET). Nevertheless, its high temporal resolution allows us to measure fast physiological processes like human brain hemodynamics. Moreover, exploiting only light, DOT is non-invasive, non-ionizing, label-free and relatively inexpensive (in particular when compared to radiology systems), thus making it extremely appealing to the brain functional imaging community.

Most of DOT systems reported in literature are based on continuous-wave technology (CW), where input light intensity is constant over time (or modulated at low frequency to get rid of 1/f noise) and light attenuation between source and detector is measured [9]. Such technology is known to be cheap, compact and easy to use. In addition, in high-density multiple-optode systems, performances comparable to fMRI were demonstrated [10]. However, CW techniques suffer from significant drawbacks. For instance, absorption and scattering are known to be natively coupled for CW technology [11]. Moreover, CW systems are strongly dependent on source-detector distances and superficial perturbations [12]. Since the use of longer source-detector separations allows us to probe deeper tissues, commercial systems exploit a combination of multiple source-detector distances to image both shallow and deep tissues, thus enabling discrimination between scalp and brain hemodynamics. However, the use of large source-detector distances affects both the signal-to-noise ratio (due to the extreme light attenuation in tissues) and the sensitivity and spatial resolution (due to the broadening of the probed region) [12]. Further, the use of large probes with high density of sources and detectors poses many difficulties for some applications such as neonate brain imaging.

An alternative option is to adopt time-domain systems (TD) [12]. Those systems are equipped with lasers that emit picosecond light pulses, and time-correlated single-photon counting (TCSPC) electronics to measure the arrival times of detected photons. The result is a histogram that represents the number of photons collected within a nanosecond-order time-frame at picosecond resolution. The idea behind TD systems is to enhance resolution in depth. This is achieved by encoding depth sensitivity via photon arrival times, e.g., a photon arrived later is more probable to have traveled deeper into the tissue [13]. In addition, since depth sensitivity is already encoded in the arrival times there is no need to construct probes with large source-detector distances [14]. Therefore, detrimental effects of the use of large distances can be avoided and smaller probes with lower number of optodes can be designed.

Nonetheless, the use of TD systems has not become widely popular mainly because of their higher cost (as compared to CW devices) and intrinsic hardware complexity. For example, most TD systems reported in the literature are based on photomultiplier tubes (PMTs) [15,16,17,18]. Such detectors are quite bulky and not scalable due to the isolation of the vacuum tube and the need of proper cooling and power supply. For that reason, the use of different solid-state detectors has also been investigated. Single photon avalanche diodes (SPADs) have been proposed [19,20] but this is not a fully satisfactory technology due to their small detection area [21], which limits the light harvesting efficiency.

Recently, the use of Silicon photomultiplier (SiPM) detectors was proposed [22]. SiPMs are solid-state detectors which possess interesting properties, namely: a wide active area and large numerical aperture, high quantum efficiency, wide spectral coverage (from 350 nm to 1000 nm), low cost, low complexity, robustness and immunity to electromagnetic fields. Additionally, they are compact and require simple front-end circuits, permitting to host them directly inside the probe, in contact with the tissue under investigation, maximizing the light collection efficiency by fully exploiting their large numerical aperture and avoiding the need for optical fibers. Due to these advantages, SiPMs have been introduced for imaging purposes (thus also for DOT) in many diffuse optics applications. For example, SiPMs have been used for CW imaging system (see, for example, Refs [23,24]) while several works show the suitability of SiPMs for DOT systems working both in frequency domain [25,26] and time domain. In the latter case, a couple of previous works described the first TD-DOT systems based on SiPM detectors [27,28]. The former system, however, was still relying on optical fibers for light collection. Instead, the latter one was based on a single injection source at 689 nm wavelength in combination with eight probe-hosted SiPM detectors [29] located at equal distances from the source and directly put in contact with the sample, thus maximizing the advantages of microelectronics devices. The system was tested both on phantoms and in vivo (on brain functional experiments). However, there were three factors that impeded to take full advantage of the proposed technology for in vivo applications: (i) the absence of multiple wavelengths, preventing the possibility to estimate oxy- and deoxy-hemoglobin concentrations [30], (ii) the use of only one injection point, decreasing the spatial sampling and thus the quality of reconstructed images and (iii) the lack of sensitivity normalization, causing the appearance of surface artifacts which prevented to accurately recover the depth location of brain activation.

In this paper, we propose a novel TD tomographic system for functional brain imaging applications, which was developed starting from the technology developed in [28]. The new system was still equipped with the same eight SiPM probe-hosted detectors, but was upgraded with six injection fibers to shine the sample in several points and with an automatic switch that permitted to perform a complete measurement in less than one second. Further, the system was provided with a dual-wavelength ( 670 nm and 820 nm) light source to quantify the changes in oxy- and deoxy-hemoglobin concentration in the tissue. Finally, the new reconstruction algorithm was based on the first 25 orders of Mellin–Laplace time-gates [31] for depth sectioning. This novel system was challenged against a solid switchable phantom experiment, and two in-vivo tests, namely arm occlusion and motor cortex activation via a finger tapping task.

The paper is organized as follows: in the second section we describe the system design and key features. In the third section, we explain the used solid phantom and the performed in-vivo tests. We also describe the details of the signal processing and reconstruction algorithms in use. In the fourth section, we discuss the results obtained and in the fifth section we summarize some conclusions on the proposed system.

## 2. System Design

**Figure 1:**
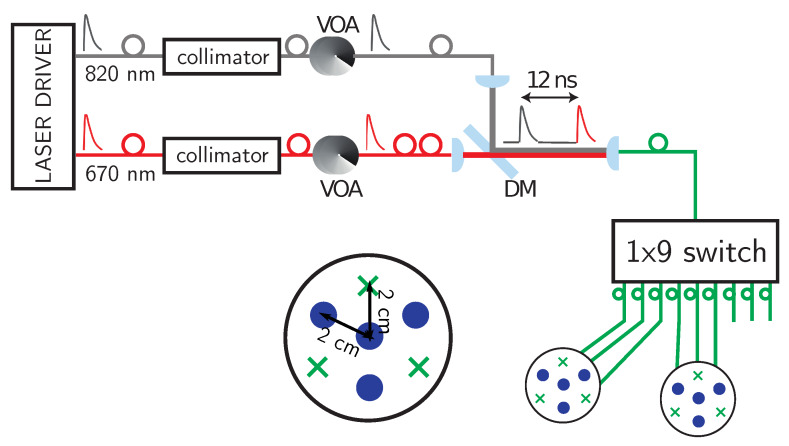
The optical system used in experiments of arm cuff occlusions and motor cortex activation. It consists of two laser beams (properly attenuated by means of a variable optical attenuator—VOA) and then combined together thanks to a dichroic mirror (DM). Light is then sequentially sent through a 1×9 switch to one of the injection fiber of two probes (each one with three fibers and four detectors). In the probes, the distance between the centered detector and the surrounding fiber inputs and detectors was 2cm.

As schematically depicted in Figure 1, the system was based on two laser diodes controlled by the same laser driver (PDL 800, Picoquant GmbH, Berlin, Germany). They provided optical pulses at 40 MHz repetition rate at two different wavelengths ( 670 nm and 820 nm). Each laser output was equipped with its own collimation stage, which also hosts the variable optical attenuator (VOA) to adjust the laser power injected into the fiber ( 100 μm core diameter). Each fiber was held on a tilting mechanical holder (for aligning purposes) and then one plano-convex lens (f = 30 mm, diameter 1 inch, Thorlabs, New Jersey, USA) was inserted to collimate the beam exiting from the fiber. To align both wavelengths onto the same path, a dichroic mirror (DM) with cut off wavelength at 805 nm (DMLP805, Thorlabs, New Jersey, USA) was inserted at 45 degrees (see Figure 1). The 820 nm beam was reflected by the dichroic mirror while the 670 nm one was transmitted and the fiber length was chosen so to separate the two wavelengths by about 12 ns delay (first: 820 nm; second: 670 nm). The free-space beam exiting from the dichroic mirror was then focused onto the 100 μm core input fiber of the 1×9 switch using the same type of plano-convex lens as before to obtain a 1:1 imaging. Driven by a microcontroller, the 1×9 switch (PiezosystemJena GmbH, Jena, Germany) continuously switches (< 30 ms switching time) the input over the nine outputs. Each of the eight outputs could be used as injection point, although in the proposed system only six of them were used (three per probe, see probes sketched in Figure 1). The switching of the output fiber was continuous and not synchronized with the acquisitions so to ensure a high speed rate needed to obtain real time tomography. Indeed, in less than one second all the six output channels were cycled, thus allowing one to perform tomographic reconstructions of the hemodynamics changes with a frame rate of > 1 Hz.

Like in reference [28], the detection chain was based on eight fiber-free SiPMs with an active area of $1.3\times 1.3\;{\mathrm{mm}}^{2}$ (S13081-050CS, Hamamatsu Photonics, Hamamatsu City, Japan). The full width at half maximum (FWHM) of the detector instrumental response function was 260ps and 360ps for 670 nm and 820 nm wavelengths, respectively. Each detector including the surrounding electronics was inserted inside a small plastic cap (for more details see [28,29]). Due to the small dimensions of the detector and its driving electronics, the eight channels could be arranged with great flexibility. In the embodiment proposed in this paper, the injection fibers and detectors were arranged in two separate probes (in each probe, there were four detectors and three injection fibers) with the same geometry as shown in Figure 1. The histogram of photon arrival times was reconstructed by an 8-channel Time-to-Digital (TDC) converter (SC-TDC-1000, Surface Concept GmbH, Mainz, Germany) featuring an average bin size of 82.2 ps. The signal synchronous with the laser synchronization pulse and the eight SiPM outputs were connected to the start channel and the eight stop channels, respectively. Since the TDC needs a Low-Voltage Transistor-Transistor Logic (LV-TTL) signal, a stack of nine custom-made signal conversion printed circuit boards to translate the analog signal to the required LV-TTL logic was designed, as explained in [32].

Since the switching of output fibers is not synchronous with the acquisition, a very short integration time ( 10 ms) was chosen to make it possible to discard in post-processing the histograms acquired while the switch was commuting. We thus acquired 16 repetitions of 10 ms (i.e., overall 160 ms) for each cycle per channel and, following the algorithm explained in Section 3.3, we discarded the repetitions corresponding to the movement (or bouncing of the tip) of the output fiber in the switch. Due to the large differential non linearity of the TDC, suitable correction algorithms have to be applied in post processing (details about the processing of the histogram generated by the TDC can be found in [28,32]).

As single-photon avalanche signals from SiPMs are pretty small (∼ 1 mV), they have to be amplified to be processed by the timing electronics [22]. This amplification stage was thus inserted as close as possible to the detector to improve the signal immunity to interferences [29]. However, as faint single-photon avalanches must be amplified by more than one order of magnitude to be detected and processed by the following timing electronics, the detection chain resulted quite sensitive to electromagnetic interferences. Although better solutions can be found by improving the performance of the front-end electronics in rejecting electromagnetic noise, aiming at a proof of concept this problem was mitigated by shielding power supply cables and by placing the main sources of interferences (e.g., the pulsed laser driver) as far as possible from the SiPMs.

Another critical point is the detector temperature as previously reported by Maira et al. [33]. Indeed, due to the absence of any thermal stabilization strategy, as soon as the SiPMs were placed in contact with the subject for in-vivo measurements they experienced a thermal drift due to the contact with the human tissue at about 37 °C. For this reason, we placed the probes (previously at room temperature) on the subject and then we waited for about 15 min before running the measurement, thus ensuring that the larger thermal drift was concluded. As explained in [29], in a time interval of 30 min the number of counts and temporal position of the pulse (center of gravity) were varying by about one percent thus enabling reliable in-vivo measurements (whose duration was about 30 min).

## 3. Experimental Protocols

In this section, we briefly describe the dynamic phantom and the protocols adopted to validate the new system both on reproducible laboratory test beds and on standard in vivo exercises. Further, the analysis and reconstruction algorithms are presented.

### 3.1. Mechanically Switchable Solid Phantom

The instrument was tested on a mechanically switchable solid inhomogeneous phantom described in detail in [34], which implements specific features of the nEUROPt protocol [35] for performance assessment of brain imagers. The phantom is composed of a homogeneous bulk with a cavity where a rod, hosting a cylindrical black inhomogeneity at 1.5 cm below the surface can move, thus mimicking localized absorption changes. The homogeneous part of the phantom was made of epoxy resin with black toner and titanium dioxide particles to add absorbing and scattering components. The black cylindrical polyvinyl chloride inclusion yielded an equivalent absorption perturbation of $\Delta \mu_{a}=0.16\;{\mathrm{cm}}^{-1}$ at 670 nm wavelength. To carry on the measurement, we made use of one of the probes of the system (three injection fibers and four SiPM detectors, see Figure 1) that was put on the top surface of the phantom.

### 3.2. In-Vivo Experiments

We performed two in-vivo experiments consisting of (1) a venous and arterial arm cuff occlusions and (2) a finger-tapping exercise with the right and left hand as described below. The experiments were conducted on healthy volunteers who gave their written informed consent to participate. The measurement protocols were approved by the Ethical Committee of Politecnico di Milano and were conducted in compliance with the Declaration of Helsinki.

#### 3.2.1. Arm Cuff Occlusion Protocols

**Figure 2:**
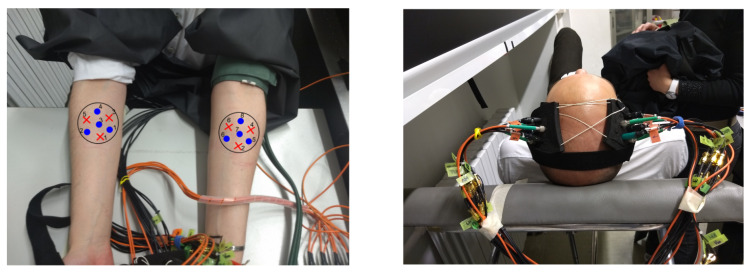
Location of probes for arm cuff occlusion (**left**) and finger tapping experiments (**right**).

We carried out two different types of arm occlusion experiments: venous and arterial occlusion. Three healthy adult subjects (one female, two male), whose average systolic and diastolic pressure were between 120–130 mmHg and 80–90 mmHg, respectively, participated in the experiment. The probes were located on the forearm of the subjects (see Figure 2 left). The occlusion was performed using a manual blood pressure cuff that was placed on the left upper arm of the subjects. The venous occlusion protocol consisted of: 60 s resting, 30 s occlusion at 100 mmHg and 90 s recovery. This protocol was repeated three times per subject. Since the cuff pressure was not increased above the diastolic pressure only veins were blocked and, therefore, only the arm output blood flow was plugged.

The arterial occlusion protocol consisted in 60 s of resting, 120 s of occlusion at 250 mmHg and 120 s of recovery; the protocol was repeated three times per subject. In this protocol, both veins and arteries were blocked which implies that arm blood inflow and outflow were totally plugged during the occlusion.

#### 3.2.2. Finger Tapping Protocol

We performed three brain motor cortex activation experiments which consisted of three different tasks: finger tapping with the left hand, finger tapping with the right hand and no tapping. The finger tapping sequence was as follows: (1) thumb touches the index finger, (2) thumb touches the ring finger, (3) thumb touches the middle finger and (4) thumb touches the pinkie finger. Three healthy adult subjects (three male, one of whom also took part in the arm occlusion experiments) volunteered for the experiment. The subjects had to repeat the sequence as fast as possible but keeping the correct order during the whole experiment. Each experiment consisted of five repetitions of 20 s resting, 20 s task and 20 s of recovery, for a total duration of 300 s. The subjects were told when to start finger tapping. In the case of not tapping the subjects did nothing during the 20 s corresponding to the task although they were also told to start finger tapping (we did so to verify the interference of any possible brain activation due to the command). In order to measure the hemodynamic changes of ipsi- and contralateral hemispheres the measurement probes were located at the C3 and C4 positions according to the 10/20 EEG international system (see Figure 2 right), with the subject lying in a comfortable bed. We waited at least 10 min before starting a new experiment with the same subject to allow for a full return to a baseline condition.

### 3.3. Data Processing

In the next subsections, we describe the data pre-processing, the analysis and the tomographic algorithm.

#### 3.3.1. Pre-Processing

**Figure 3:**
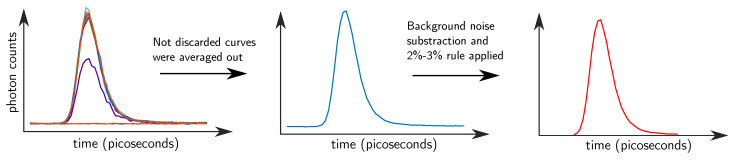
Pre-processing approach applied before analyzing the data. (1) From the 16 measured distributions of photons time-of-flight (DTOFs), the pre-switching curves were discarded. In the left image, first two curves were discarded (two noise lines at the bottom), third curve was also discarded (purple curve with low peak) and, finally, the fourth curve was still discarded to make the process more robust. (2) Not discarded curves were averaged out (middle image) and (3) the beginning and ending parts of the curves were trimmed (right image).

TDC data were pre-processed for refolding over replicas and to compensate for non-linearity [28]. Another issue to be addressed was related to the continuous asynchronous switching of the injection fiber which requires a preprocessing procedure to identify the firing source location. A cycle of the six light sources takes 0.96 s and every 10 ms a distribution of photons time-of-flight (DTOF) is acquired, therefore 16 DTOFs per fiber channel are measured. Some of those curves will bear just background noise or show a low amplitude since the switching occurred just immediately after or while they were recorded. As a result of that reason, an algorithm was designed based on the $L^{2}$ norm of the curves which detected the time-interval when the switching happened and discarded on average the first four curves from the 16 measured ones. The remaining curves were averaged (keeping distinct channels and wavelengths) and background noise was subtracted. Subsequently, the beginning and ending parts of the curves were trimmed when the number of detected photons was less than 2% and 3% of the maximum peak, respectively (see Figure 3). This processing was done to discard low signal-to-noise ratio (SNR) sections of the curves. Afterwards, to take thermal drift effects from the curves, the measurements were also detrended by subtracting any intensity increase bias.

#### 3.3.2. Intensity and Time-of-Flight Data Analysis

In this section, we describe the data analysis we performed based on standard spectroscopy data analysis procedures. With those approaches, neither spatial nor depth information is obtained but they are useful to validate the tomographic reconstruction approaches.

For arm occlusion experiments, we integrated the time-resolved curves to get the CW signal. The justification for using CW signals is that forearm muscles are very close to the surface since the fat layer is not very thick (just a few millimeters), therefore there is no need of using moments with higher sensitivity to deeper layers.

The absorption change for CW signals was recovered using the modified Beer–Lambert law,

(1) $$-\frac{1}{\langle L\rangle }\ln\left(\frac{I}{I_{0}}\right)=\Delta \mu_{a}$$

where 〈L〉 is the path length. After, the oxygenated and deoxygenated hemoglobin concentration differences were obtained using molar extinction coefficients (see, for example, the standard procedures described in [36]). Regarding motor cortex activation experiments, we decided to use the time-of-flight moment since it is more sensitive to deep layers than CW signal [37]. The absorption difference can be obtained from time-of-flight moment by using a single-layer version of Equation (15) in [13],

(2) $$\Delta \langle t\rangle =\left(\langle L\rangle \langle t\rangle -\frac{\langle L^{2}\rangle }{c}\right)\Delta \mu_{a}$$

where $\langle L^{2}\rangle =c^{2}\left(\mathrm{Var}\left(DTOF\right)-\mathrm{Var}\left(IRF\right)\right)+{\langle L\rangle }^{2}$, being *c* the speed of light in the medium, IRF the instrumental response function and $\Delta \langle t\rangle =\langle t\rangle -{\langle t\rangle }_{0}$ where ${\langle t\rangle }_{0}$ is the average time-of-flight during resting period. This baseline was re-calibrated after each task repetition.

#### 3.3.3. Mellin–Laplace Based Tomographic Algorithm

Tomographic reconstruction was performed using an algorithm based on Mellin–Laplace moments [31,38]. The algorithm was initialized by using the average optical properties estimated from fitting the DTOFs of the resting periods (before performing arm occlusion or finger-tapping). Those DTOFs were fitted to a semi-infinite half space model corrected with the instrumental response function of the system.

Regarding the discretization of the inverse problem, the sensitivity (or Jacobian) matrix obtained from Mellin–Laplace moments was weighted with a diagonal matrix. The components of the matrix were $1/\sqrt{\mathrm{Var}(M_{i})}$ where $\mathrm{Var}(M_{i})$ is the variance of the *i*-th Mellin–Laplace moment ($M_{i}$) during resting period. The motivation is to introduce into the system the uncertainty of the moments due to physiological phenomena not related to the occlusion or motor cortex activation.

Reconstruction results were regularized with a Laplacian operator. In [39], it was reported the use of Laplacian regularization to retrieve the depth of brain activity using DOT. Nevertheless, they propose to use the regularization term $∥L\delta \mu_{a}{∥}_{2}^{2}$ where *L* is the Laplacian operator. This term imposes Laplacian regularization in the absorption update, $\delta \mu_{a}$, but not in the solution itself. For this reason, in this work, we decided to use the regularizing term $∥L\left(\mu_{a}+\delta \mu_{a}\right){∥}_{2}^{2}$ that imposes a Laplacian regularized solution.

Finally, another critical aspect to deal with is the high sensitivity to shallow layers. A small absorption in surface layers has a large influence in the diffuse optical model. Therefore, the reconstruction algorithm tends to project the absorption reconstructions to the surface. To avoid this effect, the standard practice in literature is to weight deep nodes to normalize the sensitivity through all the domain: for example, using a layer-based sigmoid adjustment [40], a radial distance penalty [41,42] or a depth compensation term [43,44]. In this work, we normalized the sensitivity of nodes by weighting them with a value proportional to the distance to the surface. The proportionality hyperparameter was computed by performing reconstruction for measurements done with the switchable phantom, where the depth of the optical perturbation is known. The proportionality hyperparameter was also validated in simulations yielding accurate localization results for inclusions up to 2 cm deep.

Nevertheless, in some cases, depth normalization is not sufficient to avoid all surface artifacts. In human brain measurements, hemodynamics is constantly changing in the scalp tissue and it considerably contaminates the brain activation signals. To avoid these issues, the approach that we took in this work was to reconstruct the domain from bottom to the top surface. This method works as follows: in each iteration, reconstruction is performed only from a given depth value to the bottom. For example, for a $z_{\max}=5\;\mathrm{cm}$ cubic domain with a starting depth value of 0.4 cm depth and steps of 0.2 cm, the reconstruction iterations will be: (1) at first iteration the reconstruction is performed for nodes between 0.4 cm and 5 cm depth, (2) at second iteration the reconstruction is only performed for nodes between 0.2 cm and 5 cm, (3) at third and next iterations the reconstruction is performed in all the domain until convergence is reached. This approach forces the algorithm, during the first iterations, to perform the reconstruction at deeper layers without using superficial nodes. In the following reconstructions, an initial starting depth value of 0.8 cm was used and steps were of 0.2 cm thickness. Therefore, at the fifth iteration the algorithm starts to reconstruct the whole domain.

## 4. Results and Discussion

In this section, we first discuss the results regarding the inhomogeneous solid phantom. Then, we validate the system with the arm cuff occlusion experiments whose typical results are well-known, thus permitting to verify the capability of the instrument in reconstructing hemodynamic changes. In this case, tomographic reconstructions have not been performed as the change typically occurs in the whole arm, without localizations. Finally, we show the capabilities of the system for real-time optical tomography of brain function in the motor cortex.

### 4.1. Solid Phantom

**Figure 4:**
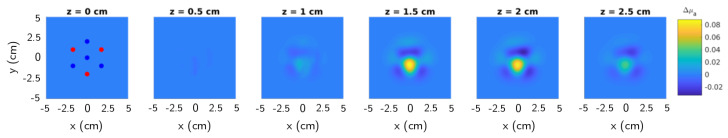
Reconstruction of a cylindrical absorbing perturbation with 5mm diameter and 5mm height located at x=(0,−1,1.5) cm depth inside a solid phantom. Perturbation flat faces were in parallel with flat faces of the cylindrical phantom. The equivalent absorption perturbation for 670 nm was $\Delta \mu_{a}=0.16\;{\mathrm{cm}}^{-1}$; for further details refer to [34].

Since the phantom does not exhibit particular spectral fingerprints, the measurements were performed at a single wavelength (670 nm). The inclusion was located at 1.5 cm depth, $\Delta \mu_{a}=0.16\;{\mathrm{cm}}^{-1}$ (for 670 nm) and it was kept still during the measurement. The reconstruction performed with Mellin–Laplace moments (25 orders and $p=12\;{\mathrm{ns}}^{-1}$) is shown at Figure 4. The reconstructed inclusion was located at the right position: between 1.5 to 2 cm depth and at y=−1cm (the probe was put in a slight off-axis position). However, the absorption difference was considerably underestimated with respect to the expected values. This phenomenon has already been described in DOT literature [45]. Therefore, the following human brain cortex activation results will be examined in terms of localization and relative hemoglobin concentration changes. However, it will not be possible to obtain absolute quantification of hemoglobin concentrations.

### 4.2. Arm Cuff Occlusion Experiments

**Figure 5:**
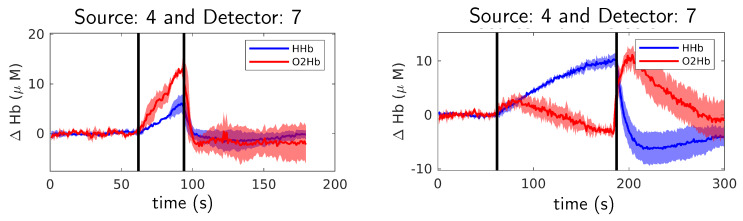
Relative oxygenated ($O_{2}\mathrm{Hb}$, red line) and deoxygenated (HHb, blue line) hemoglobin concentration for occluded arm during venous (**left**) and arterial (**right**) occlusion experiments for one of the subjects. The shadows indicate the standard deviation over three repetitions. Occlusion happened between black vertical lines.

For all arm cuff occlusion experiments, CW data analysis (see Section 3.3.2) was used to obtain the time series results. The aim of those experiments was to validate the system for physiological tests whose responses are well-known. In Figure 5 (left), the relative oxygenated and deoxygenated blood concentration time series during venous occlusion experiment is shown for one of the subjects. As expected, both oxygenated and deoxygenated blood concentration are increasing during the occlusion phase ( 60 s– 90 s) since blood inflow to the forearm is not blocked. The increase of oxygenated blood concentration is higher than deoxygenated blood because the rate of consumption of muscles is slower than the input flow. In Figure 5 (right), the results from the same subject for arterial occlusion are shown. The results are in accordance with the physiological behavior explained in Section 3.2.1. At the beginning of the occlusion, the oxygenated and deoxygenated blood increases briefly. This happens because the increase of cuff pressure to 250 mmHg value is not immediate and, therefore, at the beginning a venous occlusion takes place. This effect only happens during the first ten seconds after the occlusion starts. During the arterial occlusion both input and output blood flow are totally blocked. Therefore, the deoxygenated blood concentration increases and the oxygenated blood concentration decreases at equal rates (due to muscle oxygenated hemoglobin consumption). A few seconds after releasing cuff pressure, the concentration of oxygenated blood increases dramatically since arteries are suddenly opened. A similar phenomenon happens with deoxygenated blood but in the opposite way, that is, the concentration of deoxygenated blood dramatically decreases. After about two minutes, the concentrations of oxygenated and deoxygenated blood return to baseline values. The obtained results were very similar for the three subjects and for all fiber-detector channels (data not shown). Those results validate the capabilities of the system to reliably measure hemodynamic activity.

### 4.3. Finger Tapping Experiments

**Figure 6:**
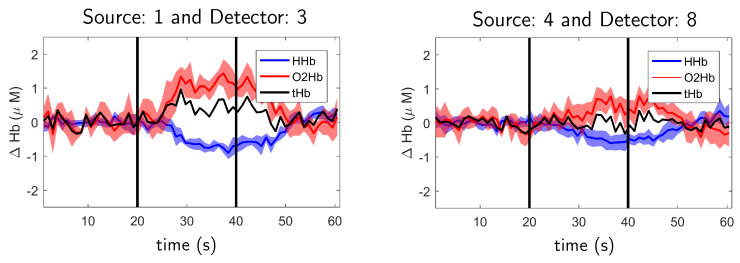
Relative oxygenated ($O_{2}\mathrm{Hb}$, red line) and deoxygenated (HHb, blue line) hemoglobin concentration for right hemisphere during left hand tapping (**left figure**) and for left hemisphere during right hand tapping (**right figure**) during finger tapping experiments for Subject 1. The shadows indicate the standard deviation over five repetitions. Black line indicates total hemoglobin. Finger tapping happened between black vertical lines. See source and detector positions for Subject 1 at Figure 7.

The expected evoked response from finger tapping implies an increase in blood flow at the motor cortex. In the response, both oxygenated hemoglobin and total hemoglobin increase due to the incrementing blood flow and volume at the motor cortex region. The physiological changes are related to the neurovascular coupling that is the activation of neurons induces blood vessels dilatation around the brain activation area [46] and, therefore, blood flow and volume is increased. The increase in the blood flow also causes deoxygenated hemoglobin to decrease, this phenomenon is known as washout effect [47]. At the same time, an increase in neural activity also induces an increase in the cerebral oxygen metabolism which converts oxygenated hemoglobin into deoxygenated. However, the increase in blood flow compensates for this effect and the net result is that blood oxygen saturation is increased in the activation area [48]. Therefore, the expected result from a motor activation is an increase of oxygenated and a decrease of deoxygenated hemoglobin. Such behavior is shown at Figure 6 for right hemisphere during left hand tapping (left figure) and for left hemisphere during right hand tapping (right figure) experiments performed by Subject 1.

Before performing the tomographic reconstructions, the data were preprocessed as explained in Section 3.3.2. Reconstructions were performed for each 0.96 s time frame, for all repetitions and for all subjects. Figure 8 displays the activation during left hand tapping for Subject 1 at t=31.7s (see the video including all time frames in Supplementary Material Video S1). The image is the result of averaging the reconstructions over the five repetitions. The first 25 orders of Mellin–Laplace moments and $p=12\;{\mathrm{ns}}^{-1}$ were used. In the image, an increase of oxygenated hemoglobin ($\Delta O_{2}\mathrm{Hb}$) and a decrease of deoxygenated hemoglobin (ΔHHb) with respect to the baseline is clearly seen in the contralateral hemisphere, as expected. The location of the activation is very close to the C4 position. In the videos included in the Supplementary Material (see Videos S2 and S3) from Subject 1 we show $\Delta O_{2}\mathrm{Hb}$ and ΔHHb concentrations separately for each repetition. The increase of $\Delta O_{2}\mathrm{Hb}$ and decrease of ΔHHb concentration is always located at the same region of the brain for the five repetitions. Moreover, the activation is always reconstructed at a depth within 1.5 to 2 cm which is the expected activation depth for an adult subject [49]. A delay of five to seven seconds can also be seen between the start of the finger tapping task and the peak increase in oxy- and deoxy-hemoglobin. This delay is in accordance with the results published in [50,51]. Quantification levels of hemoglobin concentration changes are also of the same order to data published in [30,52].

For right hand tapping, see Figure 9, activation is also seen at left hemisphere (see the Video S4 at Supplementary Material). The activation position is close to the C3 location over the tapping period for most repetitions (see Videos S5 and S6 with $\Delta O_{2}\mathrm{Hb}$ and ΔHHb concentrations for each repetition). The activation is located at similar depths such as left hand tapping. An increase of oxygenated blood is also seen at ipsilateral (right) hemisphere although there is no decrease of deoxygenated blood, for left hand tapping this ipsilateral effect could also be slightly seen. A similar phenomenon was also reported in [53]; the authors discuss that complex hand movements with dominant hand or simple hand movements with non–dominant hand tends to require more cortical activity and they also recruit some activity in the ipsilateral hemisphere. Similar conclusions are reached in [54], although they suggest that these differences disappear when subjects are overtrained.

Videos of $\Delta O_{2}\mathrm{Hb}$ and ΔHHb on both left and right hand finger tapping for Subject 2 can also be found in the Supplementary Material (see Videos S7–S10). The decrease pattern of ΔHHb at the right hemisphere during left hand tapping (Video S8) is repeated several times, although the concentration difference is very low (around ΔHHb=−0.6μM) compared to Subject 1. For this subject, the decrease of ΔHHb is located shallower than for the first subject; maxima are located at a depth between 1 to 1.5 cm.

Similar tomographic results as for Subject 2 were obtained for Subject 3. The reconstructions averaged over all repetitions do not show a decrease of ΔHHb concentration, although there is a significant increase of $\Delta O_{2}\mathrm{Hb}$ in the controlateral hemisphere for left hand tapping. However, when the results for each repetitions are shown, a common decrease of ΔHHb during finger tapping is seen (from Supplementary Material see Videos S11 and S12 for left hand tapping and Videos S13 and S14 for right hand tapping). Although, the decrease is one order of magnitude lower than $\Delta O_{2}\mathrm{Hb}$ increases, the repeatability for all repetitions is significant. The decrease of ΔHHb is located between 0.5 to 2 cm depth for left hand tapping and between 1 to 2 cm for right hand tapping.

**Figure 7:**
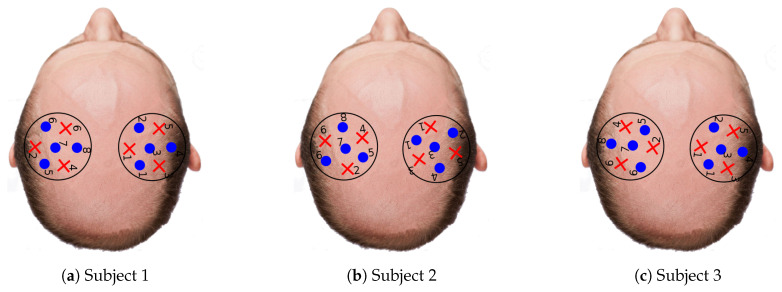
Probe locations for each of the subjects. Each probe has four detectors (blue dots) and three light fibers (red dots).

**Figure 8:**
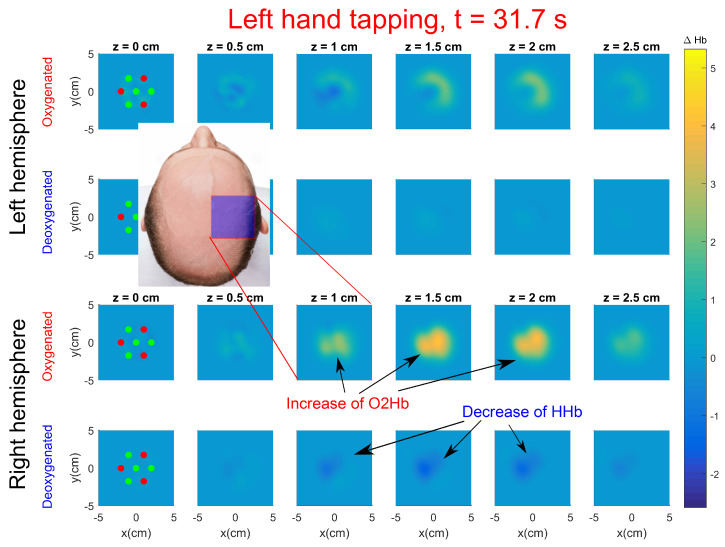
Tomographic snapshot of changes in $\Delta O_{2}\mathrm{Hb}$ and ΔHHb at right hemisphere (**bottom rows**) and left hemisphere (**top rows**) during left hand tapping performed by Subject 1. See the video including all time frames in Supplementary Material Video S1.

**Figure 9:**
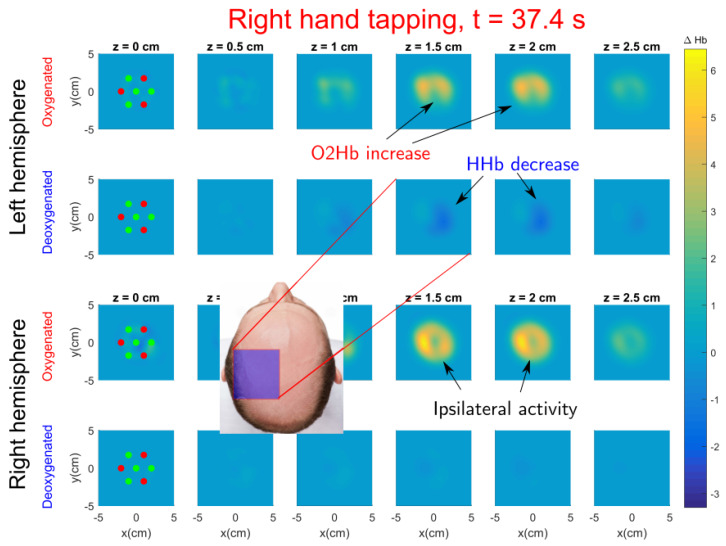
Tomographic snapshot of $\Delta O_{2}\mathrm{Hb}$ and ΔHHb left hemisphere (**top rows**) and right hemisphere (**bottom rows**) during right hand finger tapping performed by Subject 1. See the video including all time frames in Supplementary Material Video S4.

**Figure 10:**
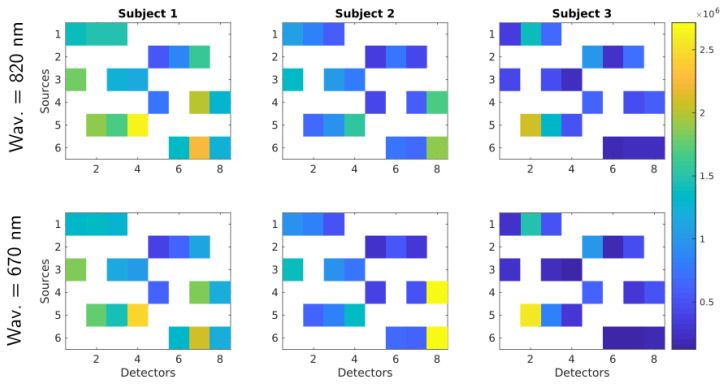
Average count rate over the three finger tapping cases. Each column represents one subject and first and second row represent 820 nm and 670 nm wavelengths, respectively. Within the matrices, rows represent fiber sources and columns detectors.

The differences in activation level among reconstructions of the three subjects can be explained due to (1) physiology reasons and (2) the count rate at the detectors. The first reason simply states that some subjects could have higher activation patterns than others. The second reason is depicted in Figure 10 where the average count rate over the three protocols for each wavelength and subject is shown (in Figure 7 the source and detector position for each subject are displayed). At first glance, it is evident that Subject 1 has a larger count rate for most channels in comparison with the rest of subjects. This was expected as Subject 1 is the unique bald subject between the three. Subjects 2 and 3 are not bald, but the hair distribution in the probed region was not homogeneous, thus giving rise to high signal only at some source-detector pairs. It is worth noting that the present system does not feature any equalization strategy at the level of the detector. The signal intensity can therefore be adjusted only by acting on the laser side with the variable attenuator. This forces the use, for each subject, of an amount of laser power that is within the single-photon statistics limit of TCSPC at the same time for all the detectors on the probes [55]. Thus, source-detectors pairs not covered by hair (yellow spots in Figure 10) affect the count rate of other pairs more covered by hair (blue spots in Figure 10). Therefore, Subjects 2 and 3 exhibit a proper count rate only for a few channels and in most cases the count rate is lower than one million counts per second. As a result of that, the measurements obtained from Subject 1 have a better signal-to-noise ratio and the tomography quality is much better.

## 5. Conclusions

In this paper, we propose a new multi-injection and dual-wavelength time-resolved system with real time (1 Hz) tomographic capability for localization of HHb and $O_{2}\mathrm{Hb}$ concentration changes. Validation of the system was provided both on a solid phantom and on arm cuff occlusion experiments. For adult human cortex activation tests, activation ($\Delta O_{2}\mathrm{Hb}$ increase and ΔHHb decrease at a depth between 1.5 to 2 cm) was clearly seen on both controlateral hemispheres for at least two subjects out of three. The tomographic system permitted to follow the evolution of the activation over time with a 1s-resolution. Problems regarding the count rate for some of the subjects were also discussed. Future work on the system will consider the improvement of the detector and its ancillary electronics addressing the present issues in signal equalization, electromagnetic interference immunity and thermal stabilization. Additionally, the recent demonstration of larger area SiPM detectors (featuring low dark count rate and good single-photon timing resolution) applied to diffuse optics applications will allow one to increase the count rate capability if applied to high-throughput TDCs, thus potentially improving the reconstruction quality. Moreover, in future upgrades, an improved design of the probe will be considered to enhance the placement and the contact between the detector and the subject head. For what concerns the measurement procedure, we are working on the synchronization between the fiber switch and the TCSPC acquisition to avoid discarding any repetition thus increasing the recorded signal per each channel.

## Supplementary Materials

The following are available online at https://zenodo.org/record/3742269. Video S1: SM_S1_averaged_L.avi; Video S2: SM_S1_O2Hb_L.avi; Video S3:SM_S1_HHb_L.avi; Video S4:SM_S1_averaged_R.avi; Video S5: SM_S1_O2Hb_R.avi; Video S6: SM_S1_HHb_R.avi; Video S7: SM_S2_O2Hb_L.avi; Video S8: SM_S2_HHb_L.avi; Video S9: SM_S2_O2Hb_R.avi; Video S10: SM_S2_HHb_R.avi; Video S11: SM_S3_O2Hb_L.avi; Video S12: SM_S3_HHb_L.avi; Video S13: SM_S3_O2Hb_R.avi; Video S14: SM_S3_HHb_R.avi. (Note: in the video title names _Sx_ stands for subject x, _L stands for left hand tapping and _R stands for right hand tapping).

## Abbreviations

The following abbreviations are used in this manuscript:

DOT	Diffuse Optical Tomography
fMRI	functional Magnetic Resonance Imaging
PET	Positron Emission Tomography
CW	Continuous–Wave
TD	Time–Domain
TCSPC	Time–Correlated Single-Photon Counting
PMT	Photomultiplier Tube
SPAD	Single-Photon Avalanche Diode
SiPM	Silicon Photomultiplier
VOA	Variable Optical Attenuator
DM	Dichroic Mirror
TDC	Time-to-Digital Converter
LV-TTL	Low-Voltage Transistor-Transistor Logic
EEG	Electroencephalography
DTOF	Distribution of photons time-of-flight
SNR	Signal-to-Noise Ratio
